# Comparative inpatient care of cancer vs. non-cancer patients in Switzerland during the national COVID-19 lockdown: a nationwide interrupted time series analysis

**DOI:** 10.1186/s12885-025-13818-5

**Published:** 2025-03-15

**Authors:** Loïc Brunner, Anna Nicolet, Isabelle Peytremann-Bridevaux, Joachim Marti, Jean-Luc Bulliard, Lorenzo Righi, Christian Britschgi, Andreas Wicki, Christine Bienvenu, Ursula Ganz-Blaettler, Manuela Eicher, Olivier Michielin, Karine Moschetti, Marie-Annick Le Pogam

**Affiliations:** 1 Department of Epidemiology and Health Systems, Center for Primary Care and Public Health (Unisanté), University of Lausanne, Lausanne, Switzerland; 2 Medical Oncology and Hematology, Cantonal Hospital Winterthur, Winterthur, Switzerland; 3 University of Zurich and University Hospital Zurich, Zurich, Switzerland; 4 Haute Ecole Arc, Jura, Switzerland; 5 SAKK Patient Advocacy Board, Berne, Switzerland; 6 Faculty of Biology and Medicine, Instiitute of Higher Education and Research in Healthcare, Lausanne University Hospital and University of Lausanne, Lausanne, Switzerland; 7 Department of Oncology, Geneva University Hospital, Geneva, Switzerland

**Keywords:** Cancer, Administrative data, ICD-10, Routinely-Collected Health Data, Chemotherapy, Radiation therapy, Hospital, Covid-19, Lockdown, Interrupted Time Series Analysis, Comparative Time Series, Switzerland

## Abstract

**Background:**

The COVID-19 pandemic exerted unprecedented pressure on healthcare systems worldwide, leading governments and hospitals to postpone elective procedures to prioritize care for COVID-19 patients. Cancer patients, who often require frequent interactions with hospital services, may have been disproportionately affected by these disuptions in healthcare delivery. This study aimed to analyze and compare hospital care provided to cancer and non-cancer inpatients during the lockdown and post-lockdown periods in Switzerland.

**Methods:**

This study analyzed comprehensive adult acute care inpatient records from national administrative hospital data spanning 2017 to 2021. Using monthly patient-level data, comparative interrupted time series and difference-in-differences analysis were conducted to assess changes in hospital care between patients with and without an ICD-10 cancer diagnosis. Changes in admission volumes, hospital outcomes (length of stay, mortality), and cancer-specific treatments (chemotherapy, radiation therapy, and palliative care) during the lockdown and post-lockdown phases of the pandemic were analyzed.

**Results:**

Hospital admissions decreased substantially during the lockdown period. From March to May 2020, non-cancer patients experienced a reduction of 17′368 admissions (-18%) (95% CI [-24′333, -10′402]), compared with a reduction of 966 admissions (-9%) (95% CI [-1′636, -296]) for cancer patients. However, despite reduction in admissions, cancer inpatients received critical treatments at rates that were no lower for chemotherapy, and 6% (95% CI [1, 12]) and 15.2% (95% CI [10; 20]) higher for radiation therapy and palliative care, respectively, during the lockdown period compared to pre-pandemic levels. The mortality rate for both groups increased during the lockdown, but the rise was 22% (95% CI [8, 32]) smaller for cancer patients compared to non-cancer patients. The length of stay increased for both groups during the lockdown. However, the difference in length of stay between cancer and non-cancer patients vanished during the lockdown, with a higher length of stay of only 0.06 days (95% CI [-0.05, 0.18]) for cancer patients, compared to 0.40 days (95% CI [0.37, 0.43]) before the lockdown.

**Conclusion:**

Swiss hospitals were able to maintain access to cancer services during the pandemic, mitigating the impact of the COVID-19 crisis for cancer population. These findings contribute to highlight the resilience of healthcare systems and understand decision-making processes during public health emergencies. However, long-term consequences of reduced care for cancer patients warrants further investigation.

**Supplementary Information:**

The online version contains supplementary material available at 10.1186/s12885-025-13818-5.

## Background

N early 2020, the COVID-19 pandemic posed unprecedented challenges for healthcare systems. The surge in critically ill patients, combined with more than 44′677 deaths and nearly 4′210′645 potential years of life lost in only 17 countries by August [1], forced healthcare providers to divert resources to COVID-19 care. To prevent health systems from collapsing, many countries, including Switzerland, implemented emergency measures such as cancelling or postponing non-urgent procedures [2–6]. While these measures were necessary, they had a significant impact on vulnerable populations, particularly cancer patients, whose care often requires time-sensitive interventions such as surgery, chemotherapy, and radiation therapy.

In Switzerland, between 2013 and 2017, an average of 42′750 new cases of cancer were diagnosed each year [7]. Cancer remained the second leading cause of death in Switzerland, accounting for 30% of deaths in men and 23% in women during this 5 years period. Most cancer diagnoses occurred in people over the age of 50, and the incidence of cancer is expected to increase by 15% in the next 7 years [8]. Cancer patients in Switzerland benefited from integrated care pathways that ensured timely diagnosis, treatment, and follow-up, although regional disparities were noted [9, 10].

In several countries, the COVID-19 pandemic affected cancer care, with a reduction in inpatient admissions during that period [11–16]. In Switzerland, hospitals faced significant pressure during the COVID-19 pandemic, as they played a crucial role in meeting the complex demands of cancer care. The federal ban on non-urgent medical procedures, imposed in March 2020 [17], could have led to delays in both cancer diagnosis and treatment, raising concerns about the potential long-term impact on cancer outcomes. Previous international studies have shown that disruptions in cancer care during the pandemic could increase cancer-related mortality. For example, the United Kingdom and the United States documented significant reductions in cancer screening and delays in treatment [18–21], while an increase in cancer mortality due to pandemic-related disruptions was projected in England, Canada, and Austria [21–23].

The specific impact of the COVID-19 pandemic on cancer care remains rather underexplored in Switzerland, especially compared to non-cancer patients where it is also underexplored worldwide. In a qualitative study examining the experiences of cancer patients during the pandemic, patients in five Swiss hospitals were interviewed and surveyed about their care during the COVID-19 period. [24]. Most patients did not report significant unmet needs or disruptions in their care, and most of them did not express particular distress related to their cancer diagnosis or fear of disease progression. This study found that the lockdown period led to a greater thickness in newly diagnosed melanomas, particularly in elderly women, and a proportional shift towards stage IV melanoma compared to the pre-lockdown [25]. This study also highlighted an increase in melanoma diagnoses immediately following the lockdown, suggesting a possible change in preventive behaviors during this period. Reversely, during the first year of the pandemic, a recent study based on cancer registry data found a decrease in all types of cancer diagnosis in the Swiss cantons of Zurich and Zug [26]. At the national level, a study focusing on a selection of inpatients procedures found a decline in elective surgeries for all types of patients during the lockdown that had not been compensated for by the end of 2020 [27]. This downward trend was also observed for elective cancer-related procedures such as mastectomies, prostatectomies, lobectomies, and colorectal resections, although the decrease was not statistically significant. Comparing the impact between hospital types, the decrease was highest for specialized hospitals and the smallest for regional hospitals. However, none of these Swiss studies compared the relative changes of the lockdown period on cancer surgery versus other procedures. The absence of national-level comparative studies between cancer care trends and other types of medical care has created a knowledge gap in the understanding of how the COVID-19 pandemic affected both cancer patients and non-cancer patients.

This study aimed to address this gap by analyzing comprehensive nationwide inpatient data from Swiss hospitals to examine the impact of containment measures undertaken during the early phases of the pandemic on the care provided to cancer and non-cancer patients. By evaluating changes in hospital admission volumes, length of stay, mortality rates, and cancer-specific treatments, this study provides insights into Swiss hospitals adaptation to the crisis and into the impact of public health measures on maintaining oncology services, and ultimately sheds light on the resilience of the healthcare system during a public health emergency.

## Methods

### Study design

This study used a comparative interrupted time series (CITS) and cross-sectional design analyzing nationwide hospital discharge data from Switzerland to assess the impact of the lockdown on key trends and outcomes before and after the COVID-19 lockdown. A before-after case–control design was used for most outcomes, with cancer patients as interests and non-cancer patients as controls, while a before-after approach was used specifically for cancer-related treatments.

### Data source

This study used national administrative hospital data from across Switzerland, provided by the Swiss Federal Statistical Office (FSO). The FSO collects standardized patient-level administrative hospital data annually, primarily for reporting, financing, and cost control purposes [28]. For the analysis, all acute care adult inpatient cases (excluding psychiatry and rehabilitation cases), with somatic stays between January 2017 and December 2021 were selected. No other selection criteria were applied.

The data included the following hospitalization information: the month of admission, 5-year age categories, sex, diagnosis according to 10th revision of the International Classification of Diseases (ICD-10) [29], treatments (chemotherapy, radiation therapy, and palliative care), length of stay (LOS) expressed in number of days, discharge mortality, readmission within 18 days of discharge, and whether the hospitalization was a planned admission. In addition, the data included information on whether intensive care was provided during the stay, and whether the stay took place at one of the five university hospitals. The primary period of interest was the COVID-19 pandemic years (2020–2021), with data from previous years (2017–2019) serving as a comparative baseline for the analysis. Reliable and complete data on COVID-19 diagnoses were not available.

### Outcomes and variables of interest

First, the study focused on the volume of hospital admissions, aggregated monthly, which provides insight into temporal patterns of hospital use. Second, it examined key outcomes, such as LOS in days, readmission rates, planned admission rates, and in-hospital deaths. Third, the analysis focused on cancer-specific treatments, examining the probability of cancer patients receiving palliative care, chemotherapy, or radiation therapy, thus highlighting patterns of specialized care.

### Statistical analyses

Data were categorized based on the COVID-19 outbreak time periods: pre-lockdown (January 2017 to February 2020), lockdown (March 2020 to May 2020), and post-lockdown (June 2020 to February 2021). Patients were further divided into adult cancer patients (aged 18 years and older) and adult non-cancer patients (aged 18 years and older). Admissions were classified as cancer-related if they contained at least one ICD-10 diagnosis code for cancer (as detailed in supplementary material 1.1), regardless of whether the code was listed as a primary or secondary diagnosis. Admissions with cancer-related ICD-10 codes were classified as cancer admissions, while those without any cancer-related codes were classified as non-cancer admissions. Cancer patients often require a range of treatments and care that go beyond cancer-specific interventions, such as management of comorbid conditions, non-cancer surgery or emergency care. By including all admissions with a cancer diagnosis—whether primary or secondary—this approach ensured that the healthcare utilization associated with their full range of needs was captured, rather than focusing only on cancer-specific admissions. This broader perspective helps to better understand the healthcare burden and resource use for cancer patients. Age was divided into three categories: under 65 years, between 65 and 80 years, and over 80 years (Supplementary material 1.1). Descriptive analyses were performed using t-tests to compare the mean differences in key variables between the pre-lockdown and lockdown or post-lockdown periods.

The main statistical analyses were carried out in three steps. First, a CITS approach was used to assess differences in the volume of hospital admissions between cancer and non-cancer patients. CITS is a well-established quasi-experimental method often used to evaluate health policies or interventions when randomization is not feasible [30]. In this study, CITS was used to examine monthly changes in admissions for cancer and non-cancer patients. The model was applied twice: first, to compare inpatient admissions between the lockdown and pre-lockdown periods, and second, to compare admissions between the post-lockdown and pre-lockdown periods. As a subgroup analysis, the CITS was also applied to older patients, female patients, patients with comorbidities (Charlson Comorbidity Index), and patients admitted to university hospitals to check for the stability of the results within subgroups of the population. Further details on the application of the model can be found in supplementary material 2.1.

Next, the impact of the pandemic and cancer status were analyzed on four outcomes: LOS, the probability of readmission within 18 days, the probability of a planned admission, and the probability of in-hospital mortality. For LOS, a negative binomial regression model was used to account for the continuous nature of the outcome. Logistic regression models were used for the binary outcomes (readmission and in-hospital death). A difference-in-differences approach was used to estimate the interaction effects of pandemic periods (lockdown and post-lockdown) and cancer status on these outcomes (Supplementary material 2.2). This method assumes parallel pre-intervention trends and allows for the estimation of multiple group-time treatment effects, providing causal estimates across both time periods [31]. Models were adjusted for time, comorbidities, age, sex, intensive care admission, and whether the admission was to a university hospital. To examine potential differences in effects by cancer stage, subgroup analyses were performed within the cancer patient group, distinguishing between metastatic and non-metastatic patients. The average of the individual marginal effects for each observation (average marginal effects), were calculated to help in the interpretation of the results. Average marginal effects show how much, on average, a one-unit change in a covariate affects the predicted dependent variable (Supplementary material 2.2).

Finally, this study focused on cancer patients to assess the impact of the lockdown on the probability of receiving specific treatments (chemotherapy, radiation therapy, and palliative care) during hospitalization. Logistic regression models were used for these binary outcomes, (Supplementary material 2.3) [32].

Statistical significance was set at p < 0.05 for all analyses. All analyses were carried out using the STATA software (Stata/SE 18.0 for Windows (64 bit × 86–64)).

## Results

### Descriptive statistics

A total of 3′958′526 hospital admissions were recorded during the study period, with a monthly average of 104′171 admissions. Among these, 92′852 were attributed to non-cancer patients and 11′319 to cancer patients. During the pre-lockdown period, the monthly average of admissions was 106′326, with 10.7% of them reporting a cancer diagnosis. During the lockdown, the percentage of cancer patients increased to 11.8%, then decreased to 11.0% in the post-lockdown period. The profiles of the cancer and non-cancer patient groups differed in terms of age, comorbidities, gender, death, LOS, planned admissions, admissions to university hospitals and use of intensive care (Table 1), and most of the differences between pre-lockdown period and the periods of interest (lockdown, from March 2020 to May 2020, and post-lockdown, from June 2020 to February 2021) were statistically significant. Generally, during the lockdown, all types of admissions decreased. However, there was a smaller decrease of cancer admissions (−966 (−9%), 95% CI [−1′636, −296]) compared to non-cancer admissions (−17′368 (−18%), 95% CI (−24′333, −10′402)). Similarly, the decrease in planned admissions was less pronounced for cancer patients (−644 (−9%); 95% CI (−1′165, 123)), than for non-cancer patients (−12′019 (−25%), 95% CI [−18′041, −5′997]). The decrease in admissions did not persist in the 9-month period after the lockdown (Table 1).

**Table 1:** Descriptive statistics of hospital admissions overall, cancer admissions and non-cancer admissions comparing the lockdown and post-lockdown periods with the pre-lockdown period

	**Pre-lockdown** **(26 months)**	**Lockdown** **(3 months)**			**Post-lockdown** **(9 months)**		
**Monthly average**	**Mean**	**Difference** **(Lock—Prelock)**		**CI 95%**	**Difference** **(Postlock—Prelock)**		**CI 95%**
**Admissions (N)**	**106′326**	**−18′334**	***−17%***	**[−25′764, −10′904]**	**−2′427**	***−2%***	**[−5′735; −882]**
Cancer (N)	11′319	−966	*−9%*	[−1′636, −296]	95	*1%*	[−273, 463]
Comorbidity > = 1 (N)	33′151	−3′411	*−10%*	[−6′384, −439]	1′833	*6%*	[483, 3′183]
Female (N)	57′337	−10′522	*−18%*	[−14′489, 6′555]	−1′795	*−3%*	[−3′622, 33]
Age > = 65 and < 80 (N)	28′061	−5′127	*−18%*	[−7′639, −2′616]	−675	*−2%*	[−1′767, 418]
Age > = 80 (N)	28′428	−4′621	*−16%*	[−6′849, −2′393]	385	*1%*	[−645, 1′414]
Intensive care (N)	6′250	−1′025	*−16%*	[−1′375, −675]	−868	*−14%*	[−1′117, −620]
Admissions within a university hospital (N)	16′868	−3′146	*−19%*	[−4′099, −2′193]	−566	*−3%*	[−1′062, −70]
Length of stay(days)	8.55	0.14	*−2%*	[−0.11, 0.39]	−0.45	*−5%*	[−0.76, −0.14]
In-hospital Death (N)	2′151	−3	*−0%*	[−239, 233]	102	*5%*	[−57, 260]
Readmissions (N)	2′929	−191	*−7%*	[−701, 320]	63	*2%*	[−194, 322]
Planned (N)	54′971	−12′663	*−23%*	[−19′026, −6′300]	−1′953	*−4%*	[−4′757, 852]
**Cancer admissions (N)**	**11′320**	**−966**	***−9%***	**[−1′636, −296]**	**95**	***1%***	**[−273, 463]**
Metastasis (N)	3531	−216	*−6%*	[−425, −7]	87	*2%*	[−35, 209]
Comorbidity > = 1 (N)	4′532	−173	*−4%*	[−561, 215]	326	*7%*	[13, 518]
Female (N)	5′103	−555	*−11%*	[−867, −244]	14	*0%*	[−156, 184]
Age > = 65 and < 80 (N)	4′738	−495	*−10%*	[−789, −200]	−66	*−1%*	[−221, 88]
Age > = 80 (N)	3′610	−320	*−9%*	[−592, −48)	259	*7%*	[120, 398]
Intensive care (N)	934	−138	*−15%*	[−204, −72]	−151	*−16%*	[−202, −100]
Admissions within a University hospital (N)	2′598	−333	*−13%*	[−523, −142]	7	*0%*	[−91, 106]
Length of stay (days)	9.40	−0.24	*−3%*	[−0.49, 0.02]	−0.62	*−7%*	[−0.89, −0.35]
In-hospital Death (N)	858	−75	*−9%*	[−131, −20]	−57	*−7%*	[−86, −29]
Readmissions (N)	251	15	*6%*	[−34, 64]	25	*10%*	[0, 50]
Planned (N)	6′869	−644	*−9%*	[−1′165, 123]	22	*0%*	[−242, 287]
**Non-Cancer admissions (N)**	**95′007**	**−17′368**	***−18%***	**[−24′333, −10′402]**	**−2′521**	***−3%***	**[−5′537, 495]**
Comorbidity > = 1 (N)	28′619	−3′238	*−11%*	[−5′893, −583]	1′507	*5%*	[320, −2′695]
Female (N)	52′234	−9′966	*−19%*	[−13′746, −6′187]	−1′809	*−3%*	[−3′504, −114]
Age > = 65 and < 80 (N)	23′322	−4′633	*−20%*	[−6′949, −2′317]	−608	*−3%*	[−1′585, 368]
Age > = 80 (N)	24′818	−4′300	*−17%*	[−6′329, −2′272]	126	*1%*	[−794, 1′045]
Intensive care (N)	5′316	−887	*−17%*	[−1′193, 581]	−718	*−14%*	[−920, 515]
Admissions within a University hospital (N)	14′270	−2′814	*−19%*	[−3′621, −2′006]	−574	*−4%*	[−986, −162]
Length of stay (days)	8.45	0.18	*2%*	[−0.07, 0.43]	−0.43	*−5%*	[−0.74, −0.12]
In-hospital Death (N)	1′293	72	*6%*	[−135, 279]	159	*12%*	[16, 302]
Readmissions (N)	2′678	−206	*−8%*	[−681, 270]	39	*1%*	[−199, 276]
Planned (N)	48′103	−12′019	*−25%*	[−18′041, −5′997]	−1′975	*−4%*	[−4′574, 624]

The table shows the averages for all outcomes for the pre-lockdown, lockdown and post-lockdown periods. The second and third parts of the table show the same averages for cancer admissions and non-cancer admissions, respectively. The 95% confidence intervals were calculated using t-tests. Prelock: pre-lockdown period, Postlock: post-lockdown period, CI 95%: 95% confidence intervals

### Volume of admissions

**Fig. 1:**
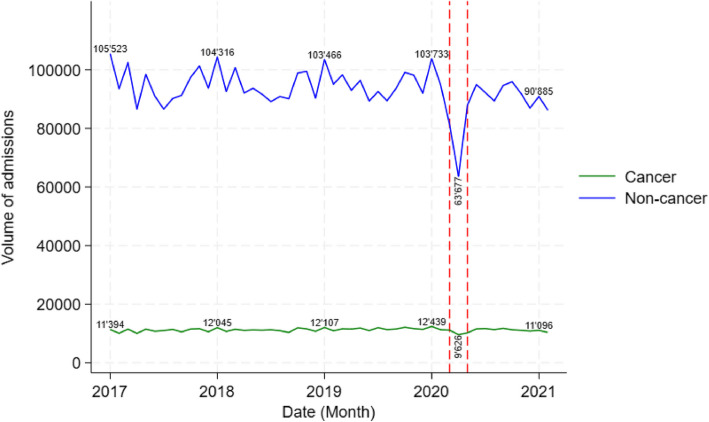
Monthly hospital admissions for cancer and non-cancer patients in Switzerland between January 2017 and February 2021. During the lockdown period, the differences in admission volumes between cancer and non-cancer patients were similarly observed within subgroups of older patients, female patients, patients with comorbidities, and those admitted to university hospitals as shown by subgroup explorations (Supplementary material 3.2). In the post-lockdown period, the difference in the reduction in admissions between cancer and non-cancer patients persisted in these subgroups but became smaller, except in the middle-aged group (65–80 years) for which no statistically significant difference in admission volume was observed between cancer and non-cancer patients

The volume of admissions decreased during the lockdown (Fig. 1) and remained lower in the post-lockdown period, than in the pre-lockdown period. We observed a statistically significant greater decrease in admissions for non-cancer patients compared to cancer patients (Supplementary material 3.1). A greater decrease in admissions was also observed for non-cancer patients with no or one comorbidity (< 2) than for those with several comorbidities (> = 2) (Supplementary material 3.3). An additional analysis accounting for cancer case indicated no statistically significant difference in admissions between metastatic and non-metastatic patients (Supplementary material 3.4).

### Length of stay, readmission, planned admissions, and in-hospital death

Cancer patients differed significantly from non-cancer patients in all outcomes. They had longer hospital stays (IRR = 1.03, 95% CI [1.03, 1.04]), lower probability of readmission (OR = 0.66, 95% CI [0.63, 0.69]), more planned admissions (OR = 2.37, 95% CI [2.34, 2.41]), and higher probability of in-hospital death (OR = 22.42, 95% CI [21.50, 23.39]).

The lockdown variable shows a significant effect across all four outcomes compared to the pre-lockdown period: an increase in LOS (IRR = 1.03, 95% CI [1.02, 1.03]), an increase in the probability of readmission (OR = 1.13, 95% CI [1.08, 1.17]), a decrease in planned admissions (OR = 0.87, 95% CI: (0.86;0.88)), and an increase in the probability of death (OR: 1.26, 95% CI [1.12, 1.41]) (Table 2).

**Table 2:** Effect of lockdown and post-lockdown periods on LOS, probability of readmission, probability of admission and in-hospital mortality for cancer patients compared with non-cancer patients

	**Length of stay (LOS)**		**Probability of readmission**		**Probability of planned admission**		**Probability of in-hospital death**	
	**IRR**	**CI 95%**	**OR**	**CI 95%**	**OR**	**CI 95%**	**OR**	**CI 95%**
Cancer	1.03***	[1.03, 1.04]	0.66***	[0.63, 0.69]	2.37***	[2.34, 2.41]	22.42***	[21.50, 23.39]
Lockdown	1.03***	[1.02, 1.03]	1.13***	[1.08, 1.17]	0.87***	[0.86, 0.88]	1.26***	[1.12, 1.41]
Post lockdown	1.02***	[1.01, 1.02]	1.01	[0.98, 1.03]	1.00	[0.99, 1.01]	1.15***	[1.07, 1.24]
Cancer X Lockdown	0.96***	[0.93, 0.98]	1.04	[0.88, 1.22]	1.20***	[1.14, 1.27]	0.78***	[0.67, 0.92]
Cancer X Post-lockdown	0.97***	[0.95, 0.98]	1.08	[0.98, 1.20]	1.09***	[1.05, 1.12]	0.85***	[0.77, 0.93]
Age 65–80	1.02***	[1.02, 1.03]	0.96***	[0.95, 0.98]	1.24***	[1.24, 1.25]	3.09***	[2.98, 3.21]
Age > 80	1.21***	[1.21, 1.22]	0.95***	[0.94, 0.97]	0.60***	[0.60, 0.61]	9.75***	[9.43, 10.07]
Comorbidities > = 1	1.35***	[1.35, 1.36]	1.90***	[1.87, 1.92]	0.59***	[0.59, 0.60]	2.63***	[2.57, 2.68]
Age 65–80 X Cancer	1.00***	[1.03, 1.05]	1.12***	[1.06, 1.19]	0.63***	[0.62, 0.64]	0.46***	[0.44, 0.48]
Age 80 X Cancer	0.958***	[0.95, 0.97]	1.26***	[1.19, 1.34]	0.83***	[0.81, 0.84]	0.20***	[0.19, 0.21]
Comorbidities > = 1 X Cancer	1.041***	[1.03, 1.05]	0.89***	[0.85, 0.93]	0.86***	[0.84, 0.87]	0.54***	[0.52, 0.55]
Age 65–80 X Lockdown	0.967***	[0.96, 0.98]	0.89***	[0.84, 0.95]	0.94***	[0.92, 0.96]	1.14*	[1.00, 1.31]
Age > 80 X Lockdown	0.901***	[0.89, 0.91]	0.88***	[0.83, 0.94]	1.04***	[1.02, 1.06]	1.084	[0.96, 1.23]
Comorbidities > = 1 X Lockdown	1.079***	[1.07, 1.09]	0.91***	[0.86, 0.96]	1.06***	[1.04, 1.08]	0.961	[0.89, 1.04]
Age 65–80 X Cancer X Lockdown	1.009	[0.98, 1.04]	0.99	[0.81, 1.21]	1.02	[0.96, 1.09]	0.89	[0.74, 1.08]
Age > 80 X Cancer X Lockdown	1.051***	[1.02, 1.09]	0.90***	[0.87, 0.93]	0.91***	[0.85, 0.97]	1.05	[0.88, 1.27]
Comorbidities > = 1 X Cancer X Lockdown	0.971**	[0.95, 1.00]	1.13	[0.96, 1.33]	0.95**	[0.90, 1.00]	1.05	[0.93, 1.18]
Age 65–80 X Post-lockdown	0.945***	[0.94, 0.95]	0.90***	[0.87, 0.93]	0.96***	[0.95, 0.97]	1.20***	[1.11, 1.31]
Age > 80 X Post-Lockdown	0.900***	[0.89, 0.91]	0.89***	[0.86, 0.93]	1.00	[0.99, 1.02]	1.164***	[1.080, 1.26]
Comorbidities > = 1 X Post-lockdown	1.076***	[1.07, 1.08]	0.93***	[0.99, 0.96]	1.05***	[1.04, 1.06]	0.98	[0.94, 1.02]
Age 65–80 X Cancer X Post-lockdown	1.030***	[1.01, 1.05]	1.05	[0.93, 1.20]	1.01	[0.97, 1.05]	0.78***	[0.70, 0.87]
Age > 80 X Cancer X Post-lockdown	1.074***	[1.05, 1.10]	0.89*	[0.78, 1.02]	0.96*	[0.92, 1.00]	0.88**	[0.79, 0.99]
Comorbidities > = 1 X Cancer X Post-lockdown	0.943***	[0.93, 0.96]	1.01	[0.92, 1.12]	0.93***	[0.90, 0.96]	1.13***	[1.05, 1.21]
Time	1.00***	[1.00, 1.00]	1.01***	[1.00, 1.01]	1.00***	[1.00, 1.00]	1.00***	[1.00, 1.00]
Female	0.992***	[0.99, 0.99]	0.77***	[0.76, 0.78]	1.00	[1.00, 1.01]	0.85***	[0.84, 0.86]
Intensive care	1.328***	[1.32, 1.33]	1.90***	[1.87, 1.93]	0.42***	[0.41, 0.42]	4.36***	[4.30, 4.43]
University hospital	0.951***	[0.95, 0.95]	0.95***	[0.94, 0.97]	0.66***	[0.66, 0.67]	1.16***	[1.14, 1.18]
lnalpha	−0.02***	[−0.02, −0.02]						
Constant	9.267***	[8.68, 9.89]	0.00***	[0.00, 0.00]	4.22***	[3.72, 4.80]	0.01***	[0.01, 0.01]
Observations	5′228′411		5′228′411		5′228′411		5′228′411	

The table shows the results of the different regressions conducted: negative binomial regression for the length of stay (LOS) and logit regression for the other outcomes. CI 95%: 95% confidence interval. IRR: incidence rate ratio, OR: odd ratio, lnalpha: the estimate of the log of the dispersion parameter of the negative binomial model, alpha. P-values: *** *p* < 0.01, ** *p* < 0.05, * *p* < 0.1

The post-lockdown variable indicates a significant increase in LOS (IRR: 1.02, 95% CI [1.01, 1.02]) and in the probability of death (OR = 1.15, 95% CI [1.07, 1.42]) when compared to the pre-lockdown period. Additionally, significant usual differences between cancer and non-cancer patients were observed before the lockdown: cancer patients had a longer LOS (IRR: 1.03, 95% CI [1.03, 1.04]), a lower probability of readmission (OR: 0.660, 95% CI [0.63, 0.96]), a higher probability of planned admission (OR: 2.37, 95% CI [2.34, 2.41]), and a higher probability of death (OR: 22.42, 95% CI [21.50, 23.39]) (Table 2).

The interaction terms showed that the observed outcome differences were not homogenous across patient groups, highlighting the nuanced impact of the lockdown (or post-lockdown), once controlled for comorbidity and age. They showed that cancer patients had a shorter LOS (IRR:0.96, 95% CI [0.93, 0.98] and IRR: 0.97, 95% CI [0.95, 0.98]), an increased probability of planned admission (OR:1.20, 95% CI [1.14, 1.27] and OR: 1.09, 95% CI [1.05, 1.12]), and a lower probability of in-hospital death (OR:0.78, 95% CI [0.67, 0.92] and OR: 0.85, 95% CI [0.77, 0.93]) in the lockdown and post-lockdown period respectively, compared with non-cancer patients and pre-lockdown period (Table 2). However, there was no significant difference between cancer and non-cancer patients in the probability of being readmitted within18 days during the lockdown period.

The subgroup analyses found no significant difference in LOS, readmissions, and probability of in-hospital death differ between metastatic and non-metastatic patients. However, it revealed a significant increase in planned admissions among metastatic patients during the post-lockdown period (Supplementary Material 3.4).

The interpretation of the effect magnitude (on average) was presented as the average marginal effects (Table 3). Compared to non-cancer patients, cancer patients stayed in the hospital 0.40 (95% CI [0.37, 0.43]) day longer in the pre-lockdown period, no statistically significant difference in the lockdown period, and 0.16 (95% CI [0.10, 0.22]) day longer in the post-lockdown period. Similarly, compared to non-cancer patients, cancer patients were 5.02% (95% CI [4.94, 5.10]) more likely to die during their hospitalization in the pre-lockdown period.This average probability decreased to 4.84% (95% CI [4.57, 5.11]) in the lockdown period and to 4.71% (95% CI [4.55, 4.87]) in the post-lockdown period. In contrast, cancer patients had 14.34% (95% CI [14.18, 14.50]) more planned admissions than non-cancer patients in the pre-lockdown period. this difference increased to 17.73% (95% CI [17.16, 18.30]) in the lockdown and 15.38% (95% CI [15.07, 15.69]) in the post-lockdown period (Table 3).

**Table 3:** Average marginal effects of having cancer diagnosis on Length of stay, Probability of readmission, Probability of planned admission and Probability of in-hospital death across the three COVID-related time periods

Outcome	Period	Cancer, dydx	CI 95%
Length of stay	Pre-lockdown	0.40	[0.37, 0.43]
	Lockdown	0.06	[−0.05, 0.18]
	Post-lockdown	0.16	[0.10, 0.22]
Probability of readmission	Pre-lockdown	−0.88%	[−0.92, −0.84]
	Lockdown	−0.75%	[−0.93, −0.57%]
	Post-lockdown	−0.69%	[−0.79, −0.59]
Probability of planned admission	Pre-lockdown	14.34%	[14.18, 14.50]
	Lockdown	17.73%	[17.16, 18.30]
	Post-lockdown	15.38%	[15.07, 15.69]
Probability of in-hospital death	Pre-lockdown	5.02%	[4.94, 5.10]
	Lockdown	4.84%	[4.57, 5.11]
	Post-lockdown	4.71%	[4.55, 4.87]

dxdy: average difference in outcome between cancer and non-cancer patients. CI 95%: 95% confidence interval

### Chemotherapy, radiation therapy, and palliative care

For cancer patients, the lockdown and post-lockdown periods were associated with an increased probability of receiving palliative care (OR = 1.15 (95% CI [1.10, 1.20]) and OR = 1.06, (95% CI [1.03, 1.09]), for both periods, respectively) and radiation therapy (OR = 1.06 (95% CI [1.01, 1.12]) for lockdown period) compared with the pre-lockdown period (Table 4). However, the probability of receiving chemotherapy did not change significantly.

**Table 4:** Effect of the lockdown-related periods (compared to pre-lockdown) on the probability of receiving palliative care, chemotherapy, or radiation therapy for cancer patients

	**Probability of receiving Chemotherapy**		**Probability of receiving Radiation therapy**		**Probability of receiving Palliative care**	
	**OR**	**CI 95%**	**OR**	**CI 95%**	**OR**	**CI 95%**
**Lockdown**	1.00	[0.96, 1.04]	1.06**	[1.01, 1.12]	1.15***	[1.10, 1.20]
**Post-Lockdown**	1.00	[0.98, 1.03]	1.02	[0.98, 1.05]	1.06***	[1.03, 1.09]
**Comorbidity (> = 1)**	1.12***	[1.10, 1.14]	0.99	[0.96, 1.01]	1.53***	[1.50, 1.56]
**Time**	0.99***	[0.99, 1.00]	1.00	[1.00, 1.00]	1.01***	[1.00, 1.01]
**Female**	0.85***	[0.84, 0.86]	0.98	[0.96, 1.01]	1.27***	[1.25, 1.30]
**Age > = 65 & < 80**	0.67***	[0.66, 0.68]	0.90***	[0.88, 0.92]	1.09***	[1.06, 1.12]
**Age > = 80**	0.35***	[0.34, 0.36]	0.69***	[0.67, 0.72]	1.11***	[1.08, 1.15]
**Big Hospital**	1.62***	[1.59, 1.64]	2.56***	[2.50, 2.62]	0.79***	[0.77, 0.81]
**Constant**	1.40**	[1.00, 1.96]	0.03***	[0.02, 0.05]	0.00***	[0.00, 0.00]
**Observations**	678′061		678′061		678′061	

This table shows the results of the logit regression. OR: odd ratio, CI 95%: 95% confidence interval. p-values: *** *p* < 0.01, ** *p* < 0.05, * *p* < 0.1

## Discussion

This study provides new insights into how Swiss hospitals managed cancer care during the COVID-19 pandemic. Specifically, it shows that cancer patients were prioritized over non-cancer patients since hospitals admissions of cancer patients decreased to a lesser extent than overall hospital admissions during the lockdown. In addition, once admitted during the lockdown, cancer patients had shorter LOS, were more likely to have planned admissions, and were less likely to die during their stay. They also appeared to receive more palliative care and radiation therapy when admitted during the COVID period.

The observed 17% reduction in total cancer hospital admissions was lower than estimates of the pandemic's impact on healthcare from other countries, which reported reductions in hospital admissions ranging from 20 to 35% in Germany, England, Scotland, Wales and New Zealand [14, 33, 34]. This difference highlights the resilience of the Swiss healthcare system. In several countries, elective procedures and non-urgent care were postponed to accommodate the surge in COVID-19 cases [11–16]. However, our study highlights a significant difference in the management of cancer versus non-cancer patients. This shows that Swiss hospitals took deliberate measures to ensure continuity of care for cancer patients, a vulnerable group of patients requiring time-sensitive treatment and care. The differences observed between cancer and non-cancer patients, showing a prioritization of care for cancer patients, were however consistent with the findings among non-cancer patients for which hospitals similarly prioritized patients with a higher number of comorbidities, underlying an emphasis on treating cases with greater medical complexity. In addition, the absence of significant differences in admission patterns between patients with and without metastases suggests that cancer cases were prioritized regardless of their cancer stage of disease.

The increase in LOS during the lockdown period, which is in line with the international literature [35], could reflect increased complexity of cases admitted during the pandemic [36–39]. Hospitals implemented stricter protocols to reduce the risk of in-hospital COVID-19 transmission, which may have contributed to increased LOS. COVID-19 may also have increased case complexity during this period, contributing to the higher average LOS. Further, the postponement of non-urgent usual procedures, which is supported by the observed higher increase in planned admission probability for cancer patients than for non-cancer patients [40], may also be a driver of the increased LOS during the lockdown period. This is further supported among cancer patients by the a higher probability of planned admission for those with metastatic disease. In addition, while overall mortality rates increased during the lockdown for both cancer and non-cancer patients, cancer patients had a relatively smaller increase in mortality, suggesting an effective management of their care, and/or a better protection of these patients from COVID-19 infection during the pandemic [41]. However, some cancer admissions were still delayed. Although these delays were less frequent than in the non-cancer population, the reduced care for cancer patients could have potentially serious long-term consequences [18–23, 42, 43].

The observed reduction in the volume of surgery, chemotherapy, and radiation therapy during the early phases of the pandemic corroborated the literature [44–50]. However, this analysis found that, once admitted, cancer patients were more likely to receive palliative care and radiation therapy than those admitted during the pre-lockdown period. The increase in the provision of palliative care and radiation therapy to cancer patients during the lockdown suggests that Swiss hospitals continued to meet the critical needs of cancer patients, particularly those requiring urgent and life-prolonging interventions.

However, despite these positive aspects, the impact of the pandemic on the wider healthcare system cannot be ignored. The large reduction in admissions of non-cancer patients and the postponement of non-urgent care are likely to bear long-term health consequences. While cancer care was prioritized, the overall burden on the healthcare system may have contributed to indirect negative outcomes for non-prioritized populations. The lasting effects of interruptions in care for other conditions have been evidenced [51, 52]. In addition, the prioritization of cancer patients may have exacerbated delays in new cancer diagnoses, potentially increasing the incidence of late-stage cancer diagnoses [53].

### Strengths and avenues for future work

This study has limitations. First, the definition of cancer patients was broad, including any patient with a cancer diagnosis according to ICD-10 codes, regardless of whether they were in active cancer treatment during the stay. This may lead to an overestimation of the number of cancer patients. Future studies may benefit from distinguishing between actively treated patients and those who have a prior history of cancer. Second, COVID-19 diagnostic data could not be analyzed, because of uncertainties about the accuracy and completeness of these data during the early phase of the pandemic. COVID-19 cases were often reported retrospectively, without systematic testing, and with no ICD-10 code for COVID-19 available in the early months of the pandemic. As a result, we were unable to distinguish the effect of the disease from the effect of the lockdown policy, as they are interrelated. Third, the study did not use a standardized comorbidity index, such as the Charlson Comorbidity Index, to account for patient health complexity. Instead, it relied on the presence of comorbidities reported during the hospital stay (Supplementary material 1.1). This approach may underestimate the burden of comorbid conditions, particularly if some relevant comorbidities are not documented. Fourth, the definition of readmission used in this study was more restrictive than in other studies that used a 30-day window instead of 18 days. Unfortunately, this was the only definition available in the dataset. It was not possible to calculate an alternative readmission indicator as the monthly data lacked the precision required to define 30-day readmissions. This shorter time frame may result in underreporting of readmissions and limits the comparability with other studies. Fifth, this study focused on inpatient care and did not include outpatient services which are critical to examine continuity of care during the lockdown, especially for chemotherapies Many outpatient services were likely postponed or shifted to telemedicine, when possible. To assess the overall impact of the lockdown, future studies should consider both inpatient and outpatient care in parallel. Future work should also aim to replicate this methodology for other types of treatments, including targeted therapies and immunotherapies. Sixth, this work compares cancer patients with non-cancer patients, without any restriction on the reason of hospitalization of non-cancer patients. The analysis was also performed within cancer patients by considering metastatic and non-metastatic patients. Results confirmed that the effects of pandemic found for metastatic and non-metastatic cancer patients were similar as those found for cancer patients versus non-cancer patients. The methodology used in the present study paves the way for future work to compare the impact of the pandemic between groups of patients with other chronic diseases that require continuous care and complex follow-up.

However, this study has several strengths. It uses comprehensive, nationwide data over an extended period of time, allowing for a robust analysis of the impact of the first phase of the COVID-19 pandemic on inpatient cancer care. The application of advanced statistical techniques to multiple outcomes and the use of subgroup analysis increased the reliability of the results. By examining both the lockdown and post-lockdown periods, this study provides a clearer understanding of how hospital practices evolved during different phases of the pandemic. The inclusion of non-cancer patients as a comparison group provides valuable context and highlights contrasts in the observed effects. Finally, this study contributes to the growing body of international evidence highlighting the importance of cross-country comparisons, as health systems have responded differently to the COVID-19 pandemic due to political and policy-related factors.

### Policy implications

This study shows how the pandemic affected cancer care and how the system adapted during an unprecedented public health emergency [54]. The results underscore the resilience of the Swiss healthcare system in meeting the needs of a vulnerable population during a global health crisis. In crisis such as COVID-19, hospitals play a key role in responding to rapid change, managing resource allocation challenges, and coping with limited capacity. As a result, hospitals should be seen as the foundation of health system resilience. Further comparative studies across regions and countries should help identify best practices for resource allocation and healthcare system flexibility to ensure effective care for both cancer and non-cancer patients in future crisis situations.

In addition, while cancer patients were prioritized during the pandemic, improved monitoring of resource allocation is essential to inform public debate on patient prioritization. Future policy should focus on improved monitoring of the adverse health outcomes associated with postponed or cancelled treatments to ensure a more equitable resource allocation and patient care. This includes assessing the long-term health effects on both cancer and non-cancer patients affected by delayed care.

To strengthen the resilience of cancer care in case of future disruptions, healthcare systems should invest in the development of guidelines and risk mitigation strategies. This could include expanding the role of healthcare professionals, such as advanced practice nurses, to mitigate potential service gaps [55]. In addition, improving communication and coordination between hospital care, primary care, and treatment services could improve the adaptability of care pathways and prioritization of patients in the future. In particular, it is important to assess the critical role of outpatient and telemedicine services during a pandemic.

## Conclusion

This study provides a comprehensive analysis of inpatient cancer care in Switzerland using administrative data from all hospitals, and valuable insights into the trade-offs between cancer and non-cancer patient care during the COVID-19 pandemic lockdown. While a decrease in cancer care was observed, it was less pronounced than for other patient groups, underscoring that Swiss hospitals successfully prioritized cancer patients during the pandemic and mitigated its most severe impacts on cancer patients. However, the reduction in non-cancer patient care admissions reflects a significant strain on the healthcare system, with potential adverse long-term consequences. It highlights the need for continued research on healthcare disruptions during crises and the importance of building resilient systems capable of managing both pandemic and routine care.

## Supplementary Information

Supplementary Material 1.

Supplementary Material 2.

Supplementary Material 3.

## Data Availability

The dataset analyzed for the current study is not publicly available due to ethical and research governance requirements for data access.

## Abbreviations

CITS: Comparative Interrupted Time Series
FSO: Federal Statistical Office
ICD-10: International Classification of Diseases
IRR: Incidence rate ratio
LOS: Length of stay
OR: Odd ratio
