# Supplementary material for: Comparative inpatient care of cancer vs. non-cancer patients in Switzerland during the national COVID-19 lockdown: a nationwide interrupted time series analysis
Source: BMC Cancer. 2025 Mar 15;25:477. doi: 10.1186/s12885-025-13818-5 (PMC11909892; doi:10.1186/s12885-025-13818-5)
Supplement: Supplementary file 1 — Supplementary Material 1. [file 12885_2025_13818_MOESM1_ESM.docx]

*Supplementary material 1: Variables and definitions*

***1.1 Variable list***

Time Periods:

- Pre-lockdown: January 2017 to February 2020 (38 months)
- Lockdown: March 2020 to May 2020 (3 months)
- Post-lockdown: June 2020 to February 2021 (9 months)

Patient Categories:

- Adult cancer patients: Hospital stays involving adult patients (age ≥ 18) with at least one cancer diagnosis according to ICD-10 (see “code use to identify variables”)).
- Adult non-cancer patients: Hospital stays involving adult patients (age ≥ 18) without any cancer diagnosis according to ICD-10 (see “code use to identify variables”)).
- Metastatic patients: Hospital stays involving adult patients (age ≥ 18 ) with at least one cancer and one metastasis diagnosis according to ICD-10 (see “code use to identify variables”) ).

Hospital Stay Characteristics:

- Length of Stay (LOS): Number of days a patient spends in the hospital during a single stay (expressed as a continuous variable in days).
- In-hospital death: Whether the patient died during the hospital stay (binary variable: 1 = death, 0 = no death).
- Readmission within 18 days: Whether the patient was readmitted within 18 days of discharge for the same diagnosis, a complication, or a retransfer (binary variable: 1 = readmission, 0 = no readmission).
- Planned admission: Whether the hospital stay was scheduled in advance for non-emergency procedures, treatment, or surgery (binary variable: 1 = planned, 0 = not planned).

Treatments:

- Palliative care: Whether the patient was treated with palliative care during the hospital stay, according to chop codes (binary variable: 1 = palliative care, 0 = no palliative care).
- Chemotherapy sessions: Whether the patient was treated with chemotherapy during the hospital stay, according to chop codes (binary variable: 1 = Chemotherapy, 0 = no chemotherapy).
- Radiation therapy: Whether the patient was treated with radiation therapy during the hospital stay, according to chop codes (binary variable: 1 = radiation therapy, 0 = no radiation therapy).

Control Variables:

- Comorbidities: Presence of one or more additional medical conditions co-occurring with the cancer condition. The comorbidities considered are acute myocardial infraction, congestive heart failure, peripheral vascular disease, cerebrovascular disease, dementia, Chronic obstructive pulmonary disease, rheumatoid disease, peptic ulcer, diabetes, diabetes with complications, hemiplegia, paraplegia, renal disease, mild liver disease, moderate liver disease, severe liver disease, and AIDS. These diseases are identified using ICD-10 codes (see “code use to identify variables”) (binary variable: 1 = one or more comorbidities, 0 = no comorbidities).
- Age of Patient: Categorical variable indicating the age group of the patient: (1: < 65 years old, 2: >= 65 & < 80 years old, and 3: >= 80 years old)
- Gender: Whether the patient is female (binary variable: 1 = female, 0 = not female).
- Intensive Care: Whether intensive care was provided during the stay (binary variable: 1 = intensive care provided, 0 = no intensive care).
- University Hospital: Whether the stay took place in a university hospital (binary variable: 1 = university hospital, 0 = non-university hospital).

Summary of Variable Types:

Binary Variables: In-hospital death, Readmission within 18 days, Planned admission, Comorbidities, Gender, Intensive Care, University Hospital, palliative care, chemotherapy, radiation therapy.

Categorical Variables: Age of Patient.

Continuous Variables: Length of Stay (LOS).

***1.2 Codes used to identify variables***

ICD-10 codes for malignant neoplasms:

- Group 1: lip, oral cavity and pharynx: "C0", "C10", "C11", "C12", "C13", and "C14"
- Group 2: digestive organs: "C15", "C16", "C17", "C18", "C19", "C20", "C21", "C22", "C23", "C24", "C25", and "C26"
- Group 3: respiratory and intrathoracic organs: "C3"
- Group 4: bones and articular cartilage: "C40", and "C41"
- Group 5: melanoma and other malignant neoplasms of skin: "C43", and "C44"
- Group 6: mesothelial and soft tissues: "C45", "C46", "C47", "C48", and "C49"
- Group 7: breast: "C50"
- Group 8: female genital organs: "C51", "C52", "C53", "C54", "C55", "C56", "C57", and "C58"
- Group 9: male genital organs: "C60", "C61", "C62", and "C63"
- Group 10: urinary tract: "C64", "C65", "C66", "C67", and "C68"
- Group 11: eye, brain and other parts of central nervous system: "C69", "C70", "C71", and "C72"
- Group 12: thyroid and other endocrine glands: "C73", "C74", and "C75"
- Group 13: lymphoid: "C8" & != "C80", and "C9" & != "C97"
- Secondary malignant neoplasms : "C78", and "C79"

Comorbidities:

- Acute myocardial infarction (AMI)I: "I21", "I22", and "I252"
- Congestive heart failure (HF): "I099", "I110", "I130", "I132", "I255", "I420", "I425", "I426", "I427", "I428", "I429", "I43", "I50", and "P290"
- Peripheral vascular disease: "I70", "I71", "I731", "I738", "I739", "I771", "I790", "I792", "K551", "K558", "K559", "Z958", and "Z959"
- Cerebrovascular disease: "G45", "G46", "I60", "I61", "I62", "I63", "I64", "I65", "I66", "I67", "I68", "I69", and "H340"
- Dementia: "F00", "F01", "F02", "F03", "G30", "F051", and "G311"
- Chronic obstructive pulmonary disease (COPD): "J40", "J41", "J42", "J43", "J44", "J45", "J46", "J47", "J60", "J61", "J62", "J63", "J64", "J65", "J66", "J67", "J684", "J701", "J703", "I278", and "I279"
- Connective tissue disease: "M05", "M06", "M32", "M33", "M34", "M315", "M351", "M353", and "M360"
- Peptic ulcer disease: "K25", "K26", "K27", and "K28"
- Mild liver disease: "B18", "K73", "K74", "K700", "K701", "K702", "K703", "K709", "K713", "K714", "K715", "K717", "K760", "K762", "K763", "K764", "K768", "K769", and "Z944"
- Diabetes mellitus uncomplicated: "E100", "E101", "E106", "E108", "E109", "E110", "E111", "E116", "E118", "E119", "E120", "E121", "E126", "E128", "E129", "E130", "E131", "E136", "E138", "E139", "E140", "E141", "E146", "E148", and "E149"
- Diabetes with end-organ damage: "E102", "E103", "E104", "E105", "E107", "E112", "E113", "E114", "E115", "E117", "E122", "E123", "E124", "E125", "E127", "E132", "E133", "E134", "E135", "E137", "E142", "E143", "E144", "E145", and "E147"
- Hemiplegia or paraplegia: "G81", "G82", "G041", "G114", "G801", "G802", "G830", "G831", "G832", "G833", "G834", and "G839"
- Moderate/severe chronic kidney disease (CKD): "N18", "N19", "I120", "I131", "N032", "N033", "N034", "N035", "N036", "N037", "N052", "N053", "N054", "N055", "N056", "N057", "N250", "Z490", "Z491", "Z492", "Z940", and "Z992"
- Moderate/severe liver disease: "I850", "I859", "I864", "I982", "K704", "K711", "K721", "K727", "K729", "K765", "K766", and "K767"
- AIDS: "B20", "B21", "B22", and "B24"
