# Supplementary material for: Comparative inpatient care of cancer vs. non-cancer patients in Switzerland during the national COVID-19 lockdown: a nationwide interrupted time series analysis
Source: BMC Cancer. 2025 Mar 15;25:477. doi: 10.1186/s12885-025-13818-5 (PMC11909892; doi:10.1186/s12885-025-13818-5)
Supplement: Supplementary file 2 — Supplementary Material 2. [file 12885_2025_13818_MOESM2_ESM.docx]

*Supplementary material 2: Method*

***2.1 Comparative interrupted time series analysis***

A comparative ITS [56, 57], is a quasi-experimental design that explores the relative changes in monthly admissions between cancer and non-cancer patients. Unlike standard ITS, which typically assess the impact of a policy change (lockdown) by comparing the outcome before and after the change while controlling for time trends, the comparative ITS extends this method by examining whether the policy impact deviate in the interest group (patient diagnosed with cancer) from the other group (patients not diagnosed with cancer) [58]. To estimate the effect of the lockdown (treatment effect), we used a generalized linear model using a regression-based approach, while also adjusting for a 12-month autocorrelation[58]. The autocorrelation was computed based on hospital discharge data spanning from January 2017 to December 2021.

The methodological approach is designed to account for count data[57]. The method is a quasi-experimental research design with a high degree of internal validity[59], offering information on the effect policy shifts that affect multiple groups simultaneously.

Given the temporary nature of the lockdown policy, we applied the model twice: once with the lockdown period and once with the post-lockdown period, each compared against the pre-lockdown period. In the first model, the post-lockdown observations are excluded, to compare only the lockdown and pre-lockdown differences. In the second model, the lockdown observations are excluded, to consider only the differences between the post and pre-lockdown periods. The two regression models used to fit these data[56] are:

$$N_{t}=\beta_{0}+\beta_{1}t+\beta_{2}{Lock}_{t}+\beta_{3}t\cdot Lock_{t}+\beta_{4}Cancer_{t}+\beta_{5}Cancer_{t}\cdot t +\beta_{6}Cancer_{t}\cdot Lock_{t}+\beta_{7}Cancer_{t}\cdot Lock_{t}\cdot t+{\beta_{8}N}_{t-12}+ \varepsilon_{t}$$

$$N_{t}=\beta_{0}+\beta_{1}t+\beta_{2}{postLock}_{t}+\beta_{3}t\cdot postLock_{t}+\beta_{4}Cancer_{t}+\beta_{5}Cancer_{t}\cdot t +\beta_{6}Cancer_{t}\cdot Lock_{t}+\beta_{7}Cancer_{t}\cdot postLock_{t}\cdot t+{\beta_{8}N}_{t-12}+ \varepsilon_{t}$$

Where $N_{t}$ is the volume of admissions, $Cancer_{t}$ is a dummy variable to denote the cancer group, t a time variable, $Lock_{t}$ and $postLock_{t}$ are dummy variables to denote the lockdown and post-lockdown. $Canc{er}_{t}\cdot t$, $Cancer_{t}\cdot Lock_{t}$, $Cancer_{t}\cdot postLock_{t}$, $Cancer_{t}\cdot Lock_{t}\cdot t$, and $Cancer_{t}\cdot postLock_{t}\cdot t$ are all interaction terms among variables. For the coefficients: $\beta_{0}$ is the baseline volume of admission for the non-cancer group; $\beta_{1}$is the pre-existing trend in volume of admissions for the non-cancer group; $\beta_{2}$is the lockdown/post-lockdown change of admission level for non-cancer group ;$\beta_{3}$is the difference in time trend of admissions during the lockdown/post-lockdown for non-cancer group;$\beta_{4}$ is the baseline difference of admissions between cancer and non-cancer groups ; $\beta_{5}$ is the baseline difference of trend in volume of admission between cancer and non-cancer groups ; $\beta_{6}$is the difference in admissions change between cancer and non-cancer groups; $\beta_{7}$ is the difference in trend change between cancer and non-cancer groups after the lockdown/post-lockdown; $\beta_{8}$is previous year’s admission volume.

***2.2 Difference in differences models***

The model used to analyze three of the outcomes (in-hospital death, readmissions, and planned admissions) is a logistic regression, which examines the hospital stays of patients individually. The model allows to investigate the effect of various predictors on binary outcomes. Logistic regression models the probability of an outcome occurring based on individual characteristics[60]. Since probability is expressed as a ratio, what is modeled is the logarithm of the odds of the outcome, given by:

$${Y_{i}}_{death}= \log\left( \frac{Prob\left( Death_{i} \right)}{1-Prob(Death_{i})} \right)$$

$${Y_{i}}_{read}= \log\left( \frac{Prob(Readmission_{i})}{1-Prob(Readmission_{i})} \right)$$

$${Y_{i}}_{planned}= \log\left( \frac{Prob\left( Planned_{i} \right)}{1-Prob(Planned_{i})} \right)$$

$${Y_{i}}_{death}=\beta_{0}+\beta_{1}Cancer_{i}+\beta_{2}Lock_{i}+\beta_{3}Post_{i}+\beta_{4}Cancer_{i}\cdot Lock_{i}+\beta_{5}Cancer_{i}\cdot Post_{i}+\beta_{j}X_{ji}+ \varepsilon_{i}$$

$${Y_{i}}_{read}=\beta_{0}+\beta_{1}Cancer_{i}+\beta_{2}Lock_{i}+\beta_{3}Post_{i}+\beta_{4}Cancer_{i}\cdot Lock_{i}+\beta_{5}Cancer_{i}\cdot Post_{i}+\beta_{j}X_{ji}+ \varepsilon_{i}$$

$${Y_{i}}_{planned}=\beta_{0}+\beta_{1}Cancer_{i}+\beta_{2}Lock_{i}+\beta_{3}Post_{i}+\beta_{4}Cancer_{i}\cdot Lock_{i}+\beta_{5}Cancer_{i}\cdot Post_{i}+\beta_{j}X_{ji}+ \varepsilon_{i}$$

Where $Prob\left( Death \right)$is the probability of in-hospital death during the stay; $Prob\left( Readmission \right)$ is the probability of readmission within the 18 days after the discharge; $Prob\left( Planned \right)$ is the probability that the stay was planned; $Lock_{i}$ is a binary variable which equals 1 if the stay of individual i started during the lockdown; $postLock_{i}$ is a binary variable which equals 1 if the stay of the individual i started during the post-lockdown period; $\beta_{0}$ is the intercept of the regression; $\beta_{1}$ is the baseline difference in outcome between cancer and non-cancer groups; $\beta_{2}$is the lockdown change of outcome level for non-cancer group; $\beta_{3}$is the post-lockdown change of outcome level for non-cancer group; $\beta_{4}$is the difference of outcome change between cancer and non-cancer groups during the lockdown; $\beta_{5}$is the difference of outcome change between cancer and non-cancer groups during the post-lockdown. $\beta_{j}$ are the coefficients of the regression associated with other adjustment variables ($X_{j}$). In the results, the table contains the exponentiated coefficients, $e^{\beta}$, which represent the odds ratios. These odds ratios indicate the change in odds associated with a one-unit change in the predictor variable.

For the LOS a negative binomial regression model is used. LOS is a count variable that represents the number of days a patient spends in the hospital. Count data often exhibit overdispersion, where the variance exceeds the mean, making a negative binomial model more appropriate than a Poisson model[61, 62]. The negative binomial model allows us to account for this overdispersion and properly estimate the effect of various predictors on LOS. The model, estimated is given by:

$${{log(Y}_{i}}_{LOS})=\beta_{0}+\beta_{1}Cancer_{i}+\beta_{2}Lock_{i}+\beta_{3}Post_{i}+\beta_{4}Cancer_{i}\cdot Lock_{i}+\beta_{5}Cancer_{i}\cdot Post_{i}+\beta_{j}X_{ji}+ \varepsilon_{i}$$

Where $Y_{i_{LOS}}$is the expected length of days for LOS, and the other elements of the equation remain the same as for logistic models.

The adjustment variables $X_{j}$ considered in the four models are:

- Comorbidities (binary variable: 1 = one or more comorbidities, 0 = no comorbidity).
- Interaction effect between lockdown and comorbidities ($Comorbidities\cdot Lock$)
- Interaction effect between post-lockdown and comorbidities $\left( Comorbidities\cdot postLock \right)$
- Age of Patient: Categorical variable (1: < 65 years old, 2: >= 65 & < 80 years old, and 3: >= 80 years old)
- Interaction effect between lockdown and age ($Age_{[65;80[}\cdot Lock$ and $Age_{\geq80}\cdot Lock$)
- Interaction effect between post-lockdown and age ($Age_{[65;80[}\cdot postLock$ and $Age_{\geq80}\cdot postLock$)
- Gender (binary variable: 1 = female, 0 = not female).
- Intensive care (binary variable: 1 = intensive care, 0 = no intensive care)
- University Hospital (binary variable: 1 = university hospital, 0 = non-university hospital).

Average marginal effects:

Non-linear models present challenges in interpreting coefficients compared to linar models. For this reason, the analysis presents Average Marginal Effects (AME), provides a solution by allowing interpretation at the population level while considering the full parameter distribution. The marginal effect for each observation was calculated using its observed values for all covariates and then averaged across the sample:

$$\frac{1}{N}\cdot\sum_{i=1}^{N} \frac{\delta E(y_{i}|{Cancer_{i}}_{i}, x_{i})}{\delta Cancer}\cdot\beta_{k}$$

Where:

- N is the total number of stays
- y is the outcome of interest
- $x$ are the covariates of the model
- $\beta_{k}$ are the parameters of interest
- $Cancer$ is the cancer binary

Note that the term average marginal effect is used in this paper for convenience, but technically it is an average incremental effect as the cancer variable is binary.

The AME are calculated at specific values to investigate the effect of the cancer binary on the 4 outcomes in different scenarios:

1. During the pre-lockdown period (lockdown = 0 and post-lockdown = 0)
2. During the lockdown period (lockdown = 1 and post-lockdown = 0)
3. During the post-lockdown period (lockdown = 0 and post-lockdown = 1)

***2.3 Logistic regressions***

The model used to analyze treatment outcomes (palliative care, chemotherapy, and radiation therapy) is a logistic regression, which examines the hospital stays of cancer patients individually to investigate the utilization of cancer treatments. The model allows to investigate the effect of various predictors on binary outcomes. Logistic regression models the probability of an outcome occurring based on individual characteristics[60]. Since probability is expressed as a ratio, what is modeled is the logarithm of the odds of the outcome, given by:

${Y_{i}}_{palliative}= \log\left( \frac{Prob\left( Palliative_{i} \right)}{1-Prob\left( Pallitive_{i} \right)} \right)=\beta_{0}+\beta_{1}Lock_{i}+\beta_{2}postLock_{i}+\beta_{j}\cdot X_{ji}+ \varepsilon_{i}$

${Y_{i}}_{chemo}= \log\left( \frac{Prob\left( Chemo_{i} \right)}{1-Prob\left( Chemo_{i} \right)} \right)=\beta_{0}+\beta_{1}Lock_{i}+\beta_{2}postLock_{i}+\beta_{j}\cdot X_{ji}{+ \varepsilon}_{i}$

$${Y_{i}}_{radio}= \log\left( \frac{Prob\left( Radio_{i} \right)}{1-Prob\left( Radio_{i} \right)} \right)=\beta_{0}+\beta_{1}Lock_{i}+\beta_{2}postLock_{i}+\beta_{j}\cdot X_{ji}+ \varepsilon_{i}$$

Where $Prob\left( Pallitive \right)$is the probability of receiving palliative care during the stay; $Prob\left( Chemo \right)$ is the probability of receiving a chemotherapy during the stay; $Prob\left( Radio \right)$ is the probability of receiving radiation therapy during the stay; $Lock_{i}$ is a binary variable which equals 1 if the stay of individual i started during the lockdown; $postLock_{i}$ is a binary variable which equals 1 if the stay of the individual i started during the post-lockdown period; $\beta_{0}$ is the intercept of the regression; $\beta_{1},\beta_{2},, \beta_{j}$ are the coefficients of the regression associated with lockdown ($Lock$), post-lockdown ($postLock$) and other adjustment variables ($X_{j}$). In the results, the table contains the exponentiated coefficients, $e^{\beta_{j}}$, which represent the odds ratios. These odds ratios indicate the change in odds associated with a one-unit change in the predictor variable. The adjustment variables $X_{j}$ considered in the models are:

- Time trend (numerical: 1, 2 ,… , increases each month)
- Comorbidities (binary variable: 1 = one or more comorbidities, 0 = no comorbidity).
- Age of Patient: Categorical variable (1: < 65 years old, 2: >= 65 & < 80 years old, and 3: >= 80 years old)
- Gender: (binary variable: 1 = female, 0 = not female).
- University Hospital: (binary variable: 1 = university hospital, 0 = non-university hospital).
