# Supplementary material for: Comparative inpatient care of cancer vs. non-cancer patients in Switzerland during the national COVID-19 lockdown: a nationwide interrupted time series analysis
Source: BMC Cancer. 2025 Mar 15;25:477. doi: 10.1186/s12885-025-13818-5 (PMC11909892; doi:10.1186/s12885-025-13818-5)
Supplement: Supplementary file 3 — Supplementary Material 3. [file 12885_2025_13818_MOESM3_ESM.docx]

*Supplementary material 3: Subgroup analysis*

***3.1 Interrupted time series analysis: volume of inpatient admissions***

Equation of the model is presented in supplementary material 2.1. The analysis confirms a significant drop in admissions during and after the lockdown period compared to the pre-lockdown period for both groups of patients. The decrease in admissions was significantly less pronounced for the cancer group than for non-cancer group, both during and after the lockdown compared to the pre-lockdown period.

**Table**: Results of the comparative time series analysis on the volume of hospital admissions. Lockdown and post-lockdown periods are compared with the pre-lockdown period.

|  | **Admissions** | | | |
| --- | --- | --- | --- | --- |
|  | **Coef** | **CI 95%** | **Coef** | **CI 95%** |
| **Time** | **57**** | [9, 105] | **30** | [-86, 146] |
| **Cancer** | **-15’555***** | [-23'840, -7’271] | **-28’382***** | [-41'432, -15’333] |
| **Time x cancer** | **-42** | [-110, 25] | **-17** | [-181, 148] |
| **Lockdown** | **-23’147***** | [-25'523, -20’772] |  |  |
| **Post lockdown** |  |  | **-5’103***** | [-8'405, -1’801] |
| **Time x lockdown** | **1’946*** | [-82, 3’974] |  |  |
| **Time x post lockdown** |  |  | **-248** | [-843, 348] |
| **Cancer x lockdown** | **21’962***** | [18'606, 25’318] |  |  |
| **Cancer x post lockdown** |  |  | **4’744**** | [75, 9’413] |
| **Time x cancer x lockdown** | **-2’611*** | [-5'476, 255] |  |  |
| **Time x cancer x post lockdown** |  |  | **121** | [-718, 960] |
| **Autocorrelation (12 months)** | **0.81***** | [0.71, 0.91] | **0.66***** | [0.50, 0.81] |
| **Constant** | **17’832***** | [8'508, 27’157] | **32’347***** | [17'755, 46’940] |
| Observations | 58 | | 70 | |
| R-squared | 0.999 | | 0.997 | |

Confidence level: *** p<0.01, ** p<0.05, * p<0.1;Coef: coefficient ; CI 95%: 95% confidence interval

***3.2 Interrupted time series analysis: within age, comorbidity, women, and university hospital***

The previous CITS analysis on admission volume is extended to subgroups of patients, including middle-aged patients (aged ≥ 65 and < 80 years), older patients (aged ≥ 80 years), patients with comorbidities, female patients, and patients admitted exclusively to university hospitals.

**Table**: Results of the comparative time series analysis on the volume of hospital admissions. Lockdown and post-lockdown periods are compared with the pre-lockdown period.

|  | **Admissions of middle aged patient (>= 65 and < 80 years old)** | | | | **Admissions of old patient (>= 80 years old)** | | | | **Admissions of comorbidity** | | | |
| --- | --- | --- | --- | --- | --- | --- | --- | --- | --- | --- | --- | --- |
|  | **Coef** | **CI 95%** | **Coef** | **IC 95%** | **Coef** | **CI 95%** | **Coef** | **IC 95%** | **Coef** | **CI 95%** | **Coef** | **CI 95%** |
| **Time** | **8** | [-8, 24] | **1** | [-35, 37] | **25**** | [6, 43] | **27** | [-21, 76] | **37**** | [5, 69] | **46** | [-14;106] |
| **Cancer** | **-2’369***** | [-3'949, -789] | **-5’462***** | [-8'097, -2’826] | **-7’093***** | [-9'290, -4’890] | **-8’166***** | [-11'883, -4’450] | **-6’645***** | [-9'762, -3’527] | **-8’996***** | [-12'888, -5’105] |
| **Time x cancer** | **-7** | [-29, 16] | **0** | [-51, 51] | **-14** | [-38, 11] | **-17** | [-85, 52] | **-28** | [-70, 14] | **-37** | [-119, 46] |
| **Lockdown** | **-6’382***** | [-7'158, -5’607] |  |  | **-6’834***** | [-7'649, -6’020] |  |  | **-7’557***** | [-8'839, -6’275] |  |  |
| **Post-lockdown** |  |  | **-1’013*** | [-2'038, 12] |  |  | **-2’065***** | [-3'338, -792] |  |  | **-2’472***** | [-3'958, -986] |
| **Time x lockdown** | **786**** | [129, 1’443] |  |  | **352** | [-316, 1’020] |  |  | **636** | [-338, 1’610] |  |  |
| **Time x Post-lockdown** |  |  | **-69** | [-254, 116] |  |  | **-120** | [-352, 111] |  |  | **-32** | [-305, 241] |
| **Cancer x lockdown** | **5’880***** | [4'786, 6’974] |  |  | **6’152***** | [5'003, 7’302] |  |  | **6’851***** | [5'053, 8’648] |  |  |
| **Cancer x Post-lockdown** |  |  | **868** | [-579, 2’315] |  |  | **1’872**** | [74, 3’670] |  |  | **2’163**** | [68, 4’258] |
| **Time x cancer x lockdown** | **-1’074**** | [-2'000, -147] |  |  | **-425** | [-1'369, 519] |  |  | **-723** | [-2'097, 653] |  |  |
| **Time x cancer x Post-lockdown** |  |  | **30** | [-231, 290] |  |  | **75** | [-253, 402] |  |  | **0** | [-386, 386] |
| **Autocorrelation (12 months)** | **0.9***** | [0.8, 0.9] | **0.7***** | [0.6, 0.8] | **0.7***** | [0.6, 0.8] | **0.6***** | [0.5, 0.8] | **0.8***** | [0.6, 0.9] | **0.7***** | (0.4, 0.8] |
| **Constant** | **3’082***** | [1'139, 5’024] | **6’941***** | [3'747, 10’135] | **8’230***** | [5'677, 10’783] | **9’500***** | [5'270, 13’731] | **7’892***** | [4'238, 11’545] | **10’697***** | [6'238, 15’156] |
| Observations | 58 | | 70 | | 58 | | 70 | | 58 | | 70 | |
| R-squared | 0.999 | | 0.995 | | 0.999 | | 0.993 | | 0.998 | | 0.993 | |

Confidence level: *** p<0.01, ** p<0.05, * p<0.1; Coef: coefficient ; CI 95%: 95% confidence interval

**Table**: Results of the comparative time series analysis on the volume of hospital admissions. Lockdown and post-lockdown periods are compared with the pre-lockdown period.

|  | **Admissions of female** | | | | **Admission of uni hosp** | | | |
| --- | --- | --- | --- | --- | --- | --- | --- | --- |
|  | **Coef** | **CI 95%** | **Coef** | **CI 95%** | **Coef** | **CI 95%** | **Coef** | **CI 95%** |
| **Time** | **23** | [-6, 52] | **8** | [-56, 73] | **11**** | [3, 20] | **6** | [-12, 25] |
| **Cancer** | **-9’400***** | [-14'715, -4’086] | **-14’971***** | [-22'511, -7’431] | **-5’236***** | [-7'789, -2’683] | **-3’975***** | [-6'511, -1’439] |
| **Time x cancer** | **-16** | [-57, 25] | **-2** | [-93, 89] | **-4** | [-16, 9] | **-0** | [-26, 26] |
| **Lockdown** | **-13’139***** | [-14'560, -11’718] |  |  | **-3’107***** | [-3'538, -2’677] |  |  |
| **Post-lockdown** |  |  | **-3’244***** | [-5'069, -1’420] |  |  | **-701***** | [-1'219, -183] |
| **Time x lockdown** | **1’278**** | [79, 2’478] |  |  | **-296** | [-657, 65] |  |  |
| **Time x Post-lockdown** |  |  | **-135** | [-463, 194] |  |  | **-167***** | [-259, -75] |
| **Cancer x lockdown** | **12’588***** | [10'580, 14’595] |  |  | **2’623***** | [2'014, 3’232] |  |  |
| **Cancer x Post-lockdown** |  |  | **3’095**** | [516, 5’675] |  |  | **503** | [-228, 1’232] |
| **Time x cancer x lockdown** | **-1’657*** | [-3'353, 38] |  |  | **183** | [-326, 693] |  |  |
| **Time x cancer x Post-lockdown** |  |  | **64** | [-399, 527] |  |  | **138**** | [7, 269] |
| **Autocorrelation (12 months)** | **0.8***** | [0.7, 0.9] | **0.7***** | [0.5, 0.8] | **0.5***** | [0.3, 0.8] | **0.7***** | [0.4, 0.9] |
| **Constant** | **10’460***** | [4'610, 16’309] | **16’619***** | [8'370, 24’868] | **6’369***** | [3'270, 9’468] | **4’838***** | [1'777, 7’898] |
| Observations | 58 | | 70 | | 58 | | 70 | |
| R-squared | 1.000 | | 0.998 | | 0.999 | | 0.997 | |

Confidence level: *** p<0.01, ** p<0.05, * p<0.1;Coef: coefficient ; CI 95%: 95% confidence interval

***3.3 Interrupted time series analysis within non-cancer patients***

To account for differences in case complexity between cancer and non-cancer patients, non-cancer patients were stratified according to their reported comorbidities. Using the previously defined comorbidity index (Supplementary Material 1.1), two groups of non-cancer patients were created: those with 0-1 comorbidity and those with multiply comorbidities (> 1 reported conditions).

The interrupted time series analysis of admissions volumes was extended to subgroups of non-cancer patients. Given the heterogeneity of non-cancer patients in terms of disease severity, analyzing specific subgroups provides more robust comparisons with cancer patients and strengthens the validity of the findings.

Next figure shows the differences in admission pattern between patients with 0-1 comorbidity and those more than 1 comorbidities. The reduction in admissions was significantly more pronounced for patients with 0-1 comorbidities compared with those with more than 1 comorbidities (see next table). These findings are consistent with the pattern observed between cancer and non-cancer patients, suggesting that hospitals moderated the impact of the Swiss lockdown policy based on case complexity.


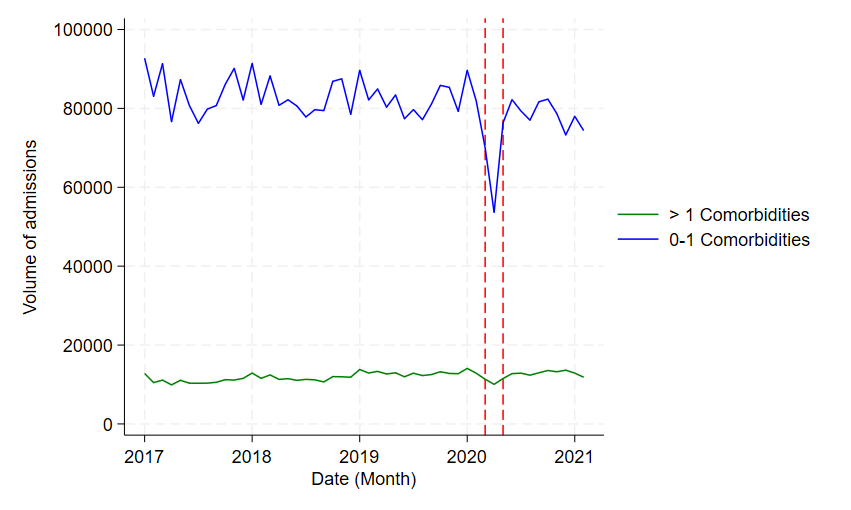


**Figure** Monthly hospital admissions for non-cancer patients with 0-1 comorbidity and non-cancer patients with > 1 comorbidities in Switzerland between January 2017 and February 2021.

**Table**: Results of the comparative time series analysis on the volume of hospital admissions of non-cancer patients, between low and high level of comorbidities.

|  | **Admissions of non-cancer patients** | | | |
| --- | --- | --- | --- | --- |
|  | **Coef** | **CI 95%** | **Coef** | **CI 95%** |
| **Time** | **37** | [-11, 85] | **3** | [-101, 106] |
| **Comorbidities > 1** | **-11’910***** | [-20’082, -3’738] | **-23,293***** | [-34’799, -11’786] |
| **Time x Comorbidities > 1** | **-17** | [-85, 51] | **24** | [-123, 171] |
| **Lockdown** | **-19’722***** | [-22'065, -17’379] |  |  |
| **Post-lockdown** |  |  | **-3’896***** | [-6’822, -970] |
| **Time x lockdown** | **1’693*** | [-300, 3’686] |  |  |
| **Time x Post-lockdown** |  |  | **-277** | [-805, 251] |
| **Comorbidities > 1 x lockdown** | **16’227***** | [2’914, 19’541] |  |  |
| **Comorbidities > 1 x Post-lockdown** |  |  | **2’747** | [-1’388, 6’883] |
| **Time x Comorbidities > 1 x lockdown** | **-1’326** | [-4’140, -4’489] |  |  |
| **Time x Comorbidities > 1 x Post-lockdown** |  |  | **300** | [-443, 1’042] |
| **Autocorrelation (12 months)** | **0.808***** | [0.698, 0.919] | **0.657***** | [0.504, 0.810] |
| **Constant** | **14’779***** | [5’481, 24’077] | **27’778***** | [4'756, 40’801] |
| Observations | 58 | | 70 | |
| R-squared | 0.999 | | 0.997 | |

Confidence level: *** p<0.01, ** p<0.05, * p<0.1; Coef: coefficient; CI 95%: 95% confidence interval

***3.4 Interrupted time series analysis within cancer patients***

To account for differences in case complexity among cancer patients, patients were stratified according to the presence or absence of metastases as documented in their admission diagnosis. Cancer patients with metastases typically represent more complex cases requiring intensive care and resources. The comparative interrupted time series analysis was then performed to compare admission patterns between metastatic and non-metastatic cancer.

The next figure shows the differences in admission patterns between cancer patients with metastases and those without metastases. The reduction in admissions for non-metastatic cancer patients is not statistically significantly different from that for patients with metastases (see next table). These findings suggest that within cancer patients, case complexity does not seem to influence the admissions of cancer patients in Switzerland.


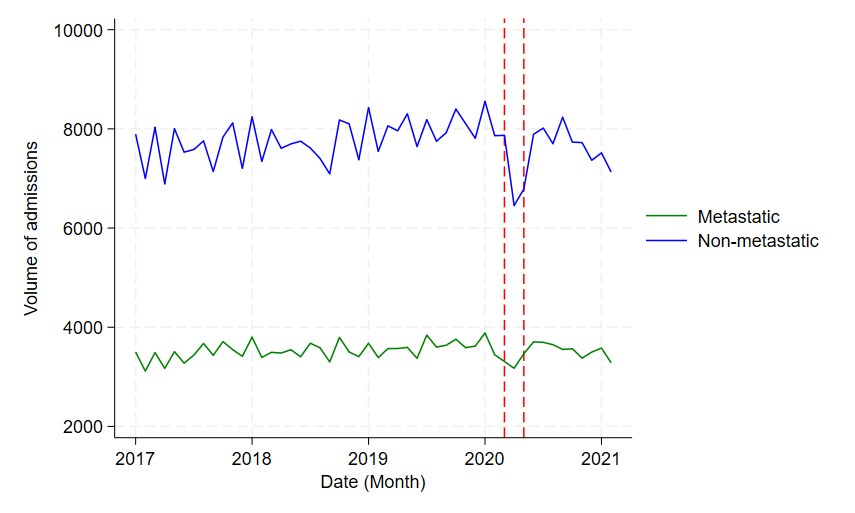


**Figure** Admission volume for cancer patient with metastases and those without metastases.

**Table**: Results of the comparative time series analysis on the volume of hospital admissions of cancer patients.

|  | **Admissions of middle-aged patient (>= 65 and < 80 years old)** | | | |
| --- | --- | --- | --- | --- |
|  | **Coef** | **CI 95%** | **Coef** | **CI 95%** |
| **Time** | **15***** | [5, 24] | **14***** | [4, 23] |
| **Metastasis** | **-1’704***** | [-2’595, -812] | **-1’801***** | [-2’536, -1’065] |
| **Time x Metastasis** | **-12**** | [-24, -0.6] | **-12*** | [-25, 0.39] |
| **Lockdown** | **-740***** | [-1’103, -377] |  |  |
| **Post-lockdown** |  |  | **-188** | [-439, 64] |
| **Time x lockdown** | **-620***** | [-898, -343] |  |  |
| **Time x Post-lockdown** |  |  | **-114***** | [-159, -69] |
| **Metastasis x lockdown** | **303** | [-210, 817] |  |  |
| **Metastasis x Post-lockdown** |  |  | **50** | [-302, 402] |
| **Time x Metastasis x lockdown** | **699***** | [309, -1’089] |  |  |
| **Time x Metastasis x Post-lockdown** |  |  | **100***** | [37, 163] |
| **Autocorrelation (12 months)** | **0.587***** | [0.375, 0.799] | **0.564***** | [0.392, 0.737] |
| **Constant** | **3’202***** | [1’603, 4’802] | **3’382***** | [2’074, 4’689] |
| Observations | 58 | | 70 | |
| R-squared | 0.996 | | 0.995 | |

Confidence level: *** p<0.01, ** p<0.05, * p<0.1; Coef: coefficient ; CI 95%: 95% confidence interval

***3.5 Difference in differences analysis between metastatic and non-metastatic cancer patients***

The model utilized for the analysis closely resembles the one outlined in the supplementary material 2.3, but it is specifically applied to a subgroup of cancer patients. Within this subgroup, a distinction is made between metastatic and non-metastatic cancer patients. The models evolved as follows:

${Y_{i}}_{read}= \log\left( \frac{Prob(Readmission_{i})}{1-Prob(Readmission_{i})} \right)$

${Y_{i}}_{planned}= \log\left( \frac{Prob\left( Planned_{i} \right)}{1-Prob(Planned_{i})} \right)$

${Y_{i}}_{death}= \log\left( \frac{Prob\left( Death_{i} \right)}{1-Prob(Death_{i})} \right)$

$${{log(Y}_{i}}_{LOS})=\beta_{0}+\beta_{1}Meta_{i}+\beta_{2}Lock_{i}+\beta_{3}Post_{i}+\beta_{4}Meta_{i}\cdot Lock_{i}+\beta_{5}Meta_{i}\cdot Post_{i}+\beta_{j}X_{ji}+ \varepsilon_{i}$$

$${Y_{i}}_{read}=\beta_{0}+\beta_{1}Meta_{i}+\beta_{2}Lock_{i}+\beta_{3}Post_{i}+\beta_{4}Meta_{i}\cdot Lock_{i}+\beta_{5}Meta_{i}\cdot Post_{i}+\beta_{j}X_{ji}+ \varepsilon_{i}$$

$${Y_{i}}_{planned}=\beta_{0}+\beta_{1}Meta_{i}+\beta_{2}Lock_{i}+\beta_{3}Post_{i}+\beta_{4}Meta_{i}\cdot Lock_{i}+\beta_{5}Meta_{i}\cdot Post_{i}+\beta_{j}X_{ji}+ \varepsilon_{i}$$

$${Y_{i}}_{death}=\beta_{0}+\beta_{1}Meta_{i}+\beta_{2}Lock_{i}+\beta_{3}Post_{i}+\beta_{4}Meta_{i}\cdot Lock_{i}+\beta_{5}Meta_{i}\cdot Post_{i}+\beta_{j}X_{ji}+ \varepsilon_{i}$$

Where $Meta_{i}$ is a binary variable which equals 1 if the stay of the individual i has a metastasis diagnosis.

**Table**: Results of the logistic and negative binomial estimations (within cancer admissions)

| **VARIABLES** | **Length of stay** | | **Probability of in-hospital death** | | **Probability of Readmission** | | **Probability of Planned admission** | |
| --- | --- | --- | --- | --- | --- | --- | --- | --- |
|  | **IRR** | **CI 95%** | **OR** | **CI 95%** | **OR** | **CI 95%** | **OR** | **CI 95%** |
| Metastasis | 1.34*** | [1.32, 1.36] | 10.33*** | [9.67, 11.02] | 1.02 | [0.92, 1.13] | 0.28*** | [0.27, 0.28] |
| Lockdown | 1.01 | [0.98, 1.03] | 0.92 | [0.73, 1.16] | 1.34*** | [1.11, 1.62] | 1.09** | [1.01, 1.16] |
| Post-lockdown | 0.99 | [0.98, 1.01] | 0.93 | [0.81, 1.07] | 1.28*** | [1.12, 1.45] | 1.06*** | [1.02, 1.11] |
| Metastasis X Lockdown | 0.97 | [0.93, 1.01] | 1.11 | [0.85, 1.44] | 0.92 | [0.65, 1.31] | 0.92 | [0.82, 1.02] |
| Metastasis X Post-lockdown | 1.01 | [0.98, 1.04] | 1.10 | [0.94, 1.29] | 0.94 | [0.75, 1.18] | 1.07** | [1.00, 1.15] |
| Age 65-80 | 1.08*** | [1.07, 1.08] | 1.74*** | [1.63, 1.85] | 1.13*** | [1.06, 1.21] | 0.82*** | [0.80, 0.84] |
| Age > 80 | 1.22*** | [1.21, 1.23] | 3.00*** | [2.82, 3.20] | 1.33*** | [1.25, 1.43] | 0.50*** | [0.48, 0.51] |
| Comorbidities >= 1 | 1.44*** | [1.43, 1.45] | 1.96*** | [1.89, 2.04] | 1.76*** | [1.67, 1.85] | 0.45*** | [0.44, 0.45] |
| Age 65-80 X Metastasis | 0.99 | [0.98, 1.09] | 0.70*** | [0.65, 0.75] | 0.87** | [0.78, 0.99] | 1.03 | [0.99, 1.06] |
| Age 80 X Metastasis | 0.94*** | [0.93, 0.96] | 0.46*** | [0.42, 0.49] | 0.68*** | [0.60, 0.77] | 1.26*** | [1.21, 1.31] |
| Comorbidities >= 1 X Metastasis | 0.87*** | [0.85, 0.88] | 0.59*** | [0.56, 0.62] | 0.95 | [0.86, 1.04] | 1.45*** | [1.41, 1.49] |
| Age 65-80 X lockdown | 0.96** | [0.93, 0.99] | 1.00 | [0.77, 1.30] | 0.85 | [0.68, 1.07] | 0.97 | [0.89, 1.05] |
| Age > 80 X Lockdown | 0.926*** | [0.90, 0.96] | 1.28* | [1.00, 1.64] | 0.76** | [0.60, 0.97] | 0.92** | [0.84, 1.00] |
| Comorbidities >= 1 X lockdown | 1.09*** | [1.06, 1.12] | 0.99 | [0.85, 1.16] | 1.05 | [0.87, 1.26] | 0.98 | [0.92, 1.05] |
| Age 65-80 X Post-lockdown | 0.98*** | [0.95, 0.99] | 0.92 | [0.77, 1.09] | 0.95 | [0.82, 1.10] | 0.99 | [0.95, 1.04] |
| Age > 80 X Post-Lockdown | 0.97*** | [0.95, 0.99] | 1.07 | [0.92, 1.24] | 0.78*** | [0.67, 0.90] | 0.97 | [0.92, 1.02] |
| Comorbidities >= 1 X Post-lockdown | 1.02*** | [1.01, 1.04] | 1.22*** | [1.11, 1.34] | 0.90* | [0.81, 1.01] | 0.99 | [0.95, 1.02] |
| Age 65-80 X Metastasis X lockdown | 1.03 | [0.98, 1.09] | 1.01 | [0.75, 1.36] | 1.12 | [0.74, 1.71] | 1.00 | [0.88, 1.14] |
| Age > 80 X Metastasis X lockdown | 1.06* | [1.00, 1.13] | 0.81 | [0.60, 1.09] | 1.30 | [0.83, 2.03] | 1.11 | [0.96, 1.28] |
| Comorbidities >= 1 X Metastasis X lockdown | 0.92*** | [0.88, 0.96] | 1.04 | [0.86, 1.27] | 0.93 | [0.66, 1.30] | 1.02 | [0.91, 1.13] |
| Age 65-80 X Metastasis X Post-lockdown | 1.02 | [0.98, 1.05] | 1.05 | [0.88, 1.26] | 0.99 | [0.76, 1.28] | 0.93* | [0.86, 1.01] |
| Age > 80 X Metastasis X Post-lockdown | 0.98 | [0.94, 1.01] | 0.92 | [0.77, 1.09] | 1.09 | [0.83, 1.45] | 1.00 | [0.92, 1.09] |
| Comorbidities >= 1 X Metastasis X Post-lockdown | 0.99 | [0.96, 1.02] | 0.88** | [0.79, 1.00] | 1.13 | [0.91, 1.39] | 0.92** | [0.87, 0.98] |
| Time | 1.00*** | [1.00, 1.00] | 0.99*** | [0.99, 1.00] | 1.00 | [1.00, 1.00] | 1.00*** | [0.99, 1.00] |
| Female | 1.07*** | [1.06, 1.07] | 0.92*** | [0.90, 0.94] | 0.80*** | [0.77, 0.83] | 1.07*** | [1.06, 1.08] |
| Intensive care | 1.91*** | [1.90, 1.93] | 1.96*** | [1.90, 2.02) | 1.60*** | [1.52, 1.69] | 0.79*** | [0.77, 0.80] |
| University hospital | 1.07*** | [1.06, 1.07] | 0.74*** | [0.72, 0.76] | 1.00 | [0.96, 1.04] | 1.10*** | [1.08, 1.11] |
| lnalpha | -0.36*** | [-0.36, -0.35] |  |  |  |  |  |  |
| Constant | 11.23*** | [9.49, 13.34] | 0.17*** | [0.08, 0.35] | 0.025*** | [0.01, 0.09] | 7.41*** | [4.91, 11.19] |
|  |  |  |  |  |  |  |  |  |
| Observations | 562’408 | | 562’408 | | 562’408 | | 562’408 | |

Confidence level: *** p<0.01, ** p<0.05, * p<0.1; Coef: coefficient ; CI 95%: 95% confidence interval; IRR: incidence rate ratio; OR: odd ratio; lnalpha: the estimate of the log of the dispersion parameter of the negative binomial model, alpha.

***3.6 Trend of admissions of patient that received palliative care, chemotherapy, and radiation therapy.***

***
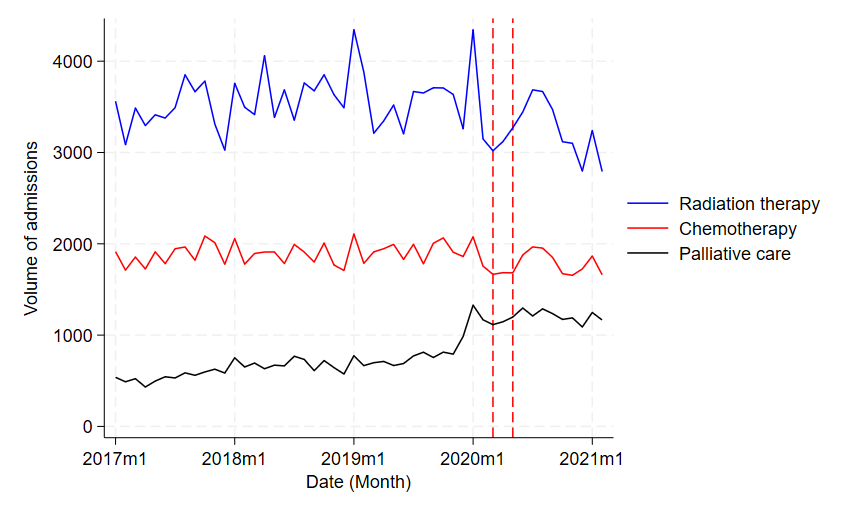
***
